# A New Amyloid‐Beta and Tau Co‐Pathology Mouse Model of Alzheimer’s Disease

**DOI:** 10.1002/alz70861_108366

**Published:** 2025-12-23

**Authors:** Anastasia Noel, Elodie Brison, Laura Griffin, Barry J Bedell

**Affiliations:** ^1^ Biospective Inc, Montreal, QC Canada; ^2^ Taconic Biosciences, Rensselaer, NY USA

## Abstract

**Background:**

Current animal models often fail to capture the complexity and key pathological features of human neurodegenerative diseases, resulting in poor translational outcomes for drug development. AAV‐mediated gene delivery in mice offers a powerful and flexible strategy to model specific aspects of human pathology *in vivo*, enabling the rapid generation of targeted disease models that are both reproducible and scalable for preclinical therapeutic screening. The goal of this work was to develop a mouse model of Alzheimer’s disease that recapitulates both amyloid‐β and tau pathologies through AAV‐mediated gene delivery, thereby enabling the study of co‐pathology mechanisms and the evaluation of therapeutic interventions in a physiologically relevant *in vivo* context.

**Method:**

Double transgenic APP/PS1 (ARTE10) or wild‐type (WT) mice at 6 months‐of‐age underwent stereotaxic injection of AAV‐hTau (wild‐type 2N4R human tau) or AAV‐null (control) vectors bilaterally into the anterior insula and the lateral entorhinal cortex. Sleep was analyzed at week 12 post‐surgery. MRI scans were acquired at weeks 4, 8, and 14, and brains were then collected at Week 14 for immunostaining analyses.

**Result:**

AAV‐Tau injection increased sleep quantity in the dark phase in the APP/PS1 mice. Extensive phosphorylated tau was observed in the neuronal soma and processes, particularly in the piriform and entorhinal cortices compared to the anterior brain regions, alongside fibrillar amyloid‐β pathology characterized by plaques and vascular deposits. Significant neuroinflammation was observed in the APP/PS1/hTau mice, in brain regions with extensive pathology. Injection of AAV‐hTau induced significant reductions in regional brain volumes and cortical thickness in both WT and APP/PS1 mice, with APP/PS1/hTau animals showing even greater cortical thinning compared to WT/hTau mice.

**Conclusion:**

This novel APP/PS1/hTau mouse model reproduces key Alzheimer’s disease features, including parenchymal and vascular amyloid‐β deposits, phosphorylated tau, microgliosis, astrogliosis, and sleep disturbances. It also exhibits a clear neurodegenerative phenotype with regional brain atrophy detectable by MRI, offering translational relevance. The model enables investigation of proteinopathy–neuroinflammation interactions and provides quantifiable in‐life and post‐mortem endpoints, making it a valuable tool for preclinical testing of disease‐modifying therapies in Alzheimer’s disease.

